# PIP_2_ activation of the cardiac I_Ks_ potassium channel

**DOI:** 10.21203/rs.3.rs-7609003/v1

**Published:** 2025-10-21

**Authors:** Jianmin Cui, Lu Zhao, Xianjin Xu, Chenxi Cui, Rui Duan, Ali Kermani, Jingyi Shi, Lu Han, Ji Sun, Xiaoqin Zou

**Affiliations:** Washington University in Saint Louis; Washington University in St. Louis; University of Missouri – Columbia; National University of Singapore; University of Missouri; St Jude Children’s Research Hospital; Washington University in St. Louis; Washington University in Saint Louis; St Jude Children’s Rsearch Hospital; University of Missouri-Columbia

**Keywords:** V-PIP2, C-PIP2, KCNQ1, IKs, anti-arrhythmia therapy

## Abstract

The I_Ks_ channel complex, composed of the voltage-gated potassium channel KCNQ1 and its regulatory subunit KCNE1, is essential for the termination of cardiac action potentials. The function of KCNQ1 and I_Ks_ requires PIP_2_, and its depletion abolishes channel opening. Previous studies revealed that KCNQ1 adopts both bent and straight conformations and can bind two PIP_2_ molecules: one adjacent to VSD (V-PIP_2_), and the other at the VSD-pore interface (C-PIP_2_). Here we show that the two PIP_2_ perform essential yet distinct roles: V-PIP_2_ enables the bent-to-straight transition, whereas C-PIP_2_ mediates VSD-pore coupling and stabilizes the straight conformation. Structure-function analysis and molecular dynamic simulations show that VSD activation elevates the V-PIP_2_ site and weakens the CaM-VSD interaction, permitting the conformational shift from the bent, intermediate open (IO) state associated with KCNQ1 to the straight, I_Ks_-exclusive activated open (AO) state, which is further stabilized by C-PIP_2_. Leveraging this mechanism, we developed a compound CA1, which selectively targets the V-PIP_2_ site and modulates I_Ks_ channel activity without affecting KCNQ1, offering a novel and promising conceptional path for specific and safe antiarrhythmic therapeutics.

Phosphatidylinositol 4,5-bisphosphate (PIP_2_) is an important signaling molecule that regulates the function of a variety of membrane proteins ^1–3^ including many of ion channels ^4,5^. Among PIP_2_-regulated ion channels, the family of KCNQ1–5 potassium (K^+^) channels plays a crucial role in regulating membrane excitability in the brain, heart and epithelium ^6–9^. PIP_2_ is required for KCNQ channel function, and channel activity diminishes when membrane PIP_2_ levels are reduced ^7,10,11^. Thus, investigating PIP_2_-dependent activation of KCNQ channels could yield valuable insights into the physiological effects and molecular mechanisms of PIP_2_ regulation in ion channels. We recently solved the structure of KCNQ1 in association with its regulatory subunit, KCNE1, and PIP_2_ molecules (**Fig. S1b**) ^11^, providing a structural foundations for understanding mechanisms of the PIP_2_-dependent activation of KCNQ1 channels.

KCNQ1 channels are voltage-gated K^+^ (Kv) channels ^12–14^. KCNQ1, together with the regulatory subunit KCNE1, forms the slow-delayed rectifier potassium (I_Ks_) channels in the heart and inner ear, which are crucial for regulating heart rhythm and supporting K^+^ recycling in the endolymph ^15–17^. When associated with the regulatory subunit KCNE3, KCNQ1 is constitutively active at physiological voltages, functioning as a K^+^ transporter to maintain ionic homeostasis in epithelial and endothelial cells ^18,19^. Malfunctions of KCNQ1 caused by drugs, mutations, and single nucleotide polymorphisms are linked to arrhythmia, deafness, atrial fibrillation and type-2 diabetes mellitus ^12,20–22^.

The voltage-dependent gating of K_v_ channels involves three fundamental processes, voltage sensor domain (VSD) activation, VSD-pore coupling, and pore opening. The VSD of Kv channels, such as Shaker and KCNQ1, can exist in three resolvable states: resting (R), intermediate (I) and activated (A). Upon membrane depolarization the VSD of these channels transition from the R state to the A state via the I state ^23–25^. However, in Shaker channels, the pore conducts only at the activated open (AO) state when the VSD is in the A state ^23,26^. In contrast, in KCNQ1, the pore opens when the VSD is in either the I or the A state, resulting in the IO (intermediate open) and AO states ^14,24,27,28^ (**Fig. S1a**). The IO and AO states exhibit drastically different properties in their voltage dependence, activation kinetics, current amplitude, ion permeability, and pharmacology ^13,14,24^.

The IO and AO states of KCNQ1 are differentially regulated by various KCNE subunits, which exhibit distinct tissue distributions ^15,16,18,19^. This differential regulation enables KCNQ1 + KCNE channels to function across diverse tissues, supporting a wide range of physiological functions. For instance, both KCNE1 and KCNE3 selectively shift the VSD activation to the I state at more negative voltages ^18^. However, KCNE1 suppresses IO but enhances AO of KCNQ1, making the channel open exclusively in the AO state, with a more positively shifted voltage dependence, slower activation kinetics, and larger current amplitudes ^24,28^. These features are essential for terminating cardiac action potential. In contrast, KCNE3 does not suppress IO, allowing KCNQ1 + KCNE3 channels open at more negative voltages and remain constitutively open at physiological voltages to maintain K^+^ homeostasis in the epithelium ^18,19^.

The structural basis underlying the IO and AO states in KCNQ1 remains unclear. KCNQ1 channels adopt a homo-tetrameric assembly, where each subunit contains six transmembrane α-helices S1-S6, with S1-S4 forming the VSD and S5 and S6 forming the central pore ^29,30^. Each KCNQ1 subunit interacts with a calmodulin (CaM) molecule mainly through its cytosolic helix A (HA) and helix B (HB) (**Fig. S1b**). Our previous structural studies revealed two distinct conformations of the cytosolic domain in KCNQ1 channels; straight and bent ^29,30^. In the straight conformation, HA forms a continuous helix with the transmembrane helix S6, positioning CaM deep in the cytosol, away from the membrane-spanning domain of KCNQ1. The bent conformation is facilitated by a structural kink at the “RQKH” motif, located between helix S6 and HA. This kink allows HA to adopt an upward-bent orientation, enabling CaM to undergo rotational displacement to interact with the cytosolic S2-S3 linker (S2S3L). Our previous functional studies demonstrated that mutations disrupting the S2S3L-CaM interface shift KCNQ1 channel properties from predominantly IO to characteristics resembling the AO state. Based on these findings, we proposed that the IO state likely adopts the bent conformation, while the AO state corresponds to the straight conformation, and that disrupting S2S3L-CaM interactions promotes the transition from the IO to the AO state ^31^.

Our prior studies demonstrated that PIP_2_ is required for VSD-pore coupling in KCNQ1 in both the IO and AO states ^7,10^. Without PIP_2_, the VSD activates but fails to induce pore opening. Functional and mutagenesis studies indicated a PIP_2_ binding site at the interface between the VSD and the pore, essential for KCNQ1 channel opening ^7^. However, our previous structural analyses of KCNQ1 and KCNQ1 + KCNE3 did not clearly show PIP_2_ binding at the VSD-pore interface but instead revealed a PIP_2_-binding site adjacent to the VSD ^30^. We recently solved the structure of the KCNQ1-KCNE1 complex, which revealed not only the PIP_2_-binding site near the VSD, but also an additional PIP_2_ site at the VSD-pore interface, yielding a total of eight PIP_2_ molecules in the channel complex ^11^ (**Fig. S1b**).

In this study we solved the structure of KCNQ1 in an I state. We examined the structural and functional properties of the two PIP_2_-binding sites in KCNQ1. These results, together with the identification of a compound, CA1, that selectively binds to one of PIP_2_ sites and specifically enhances the activation of KCNQ1 only in the presence of KCNE1, support that the IO and AO states adopt the bent and straight conformations, respectively. Through electrophysiological experiments and structural characterization of KCNQ1 channel at the I state, we found that the first PIP_2_ molecule located near the VSD and termed V-PIP_2_ for its role in VSD function (**Fig. S1b**), drives the conformational shift from IO to AO during VSD activation. VSD activation to the A state alters the conformation of V-PIP_2_ and its binding site (Fig. 4a), detaching CaM from the VSD and promoting the transition from bent to straight. The second PIP_2_ molecule, designated C-PIP_2_, was revealed in our recently determined KCNQ1-KCNE1 structure at the VSD-pore interface, promoting VSD-pore coupling to enable channel opening in both IO and AO states while stabilizing the straight conformation (Figs. 4g, **S1b**) ^11^. VSD-pore coupling determines the channel’s ability to open, whereas the IO-to-AO transition is critical for the function of I_Ks_ channels in the heart. Therefore, both PIP_2_ molecules are vital for the physiological functions of KCNQ1 channels.

## Conformations of KCNQ1 in various functional states

**Figure 1:**
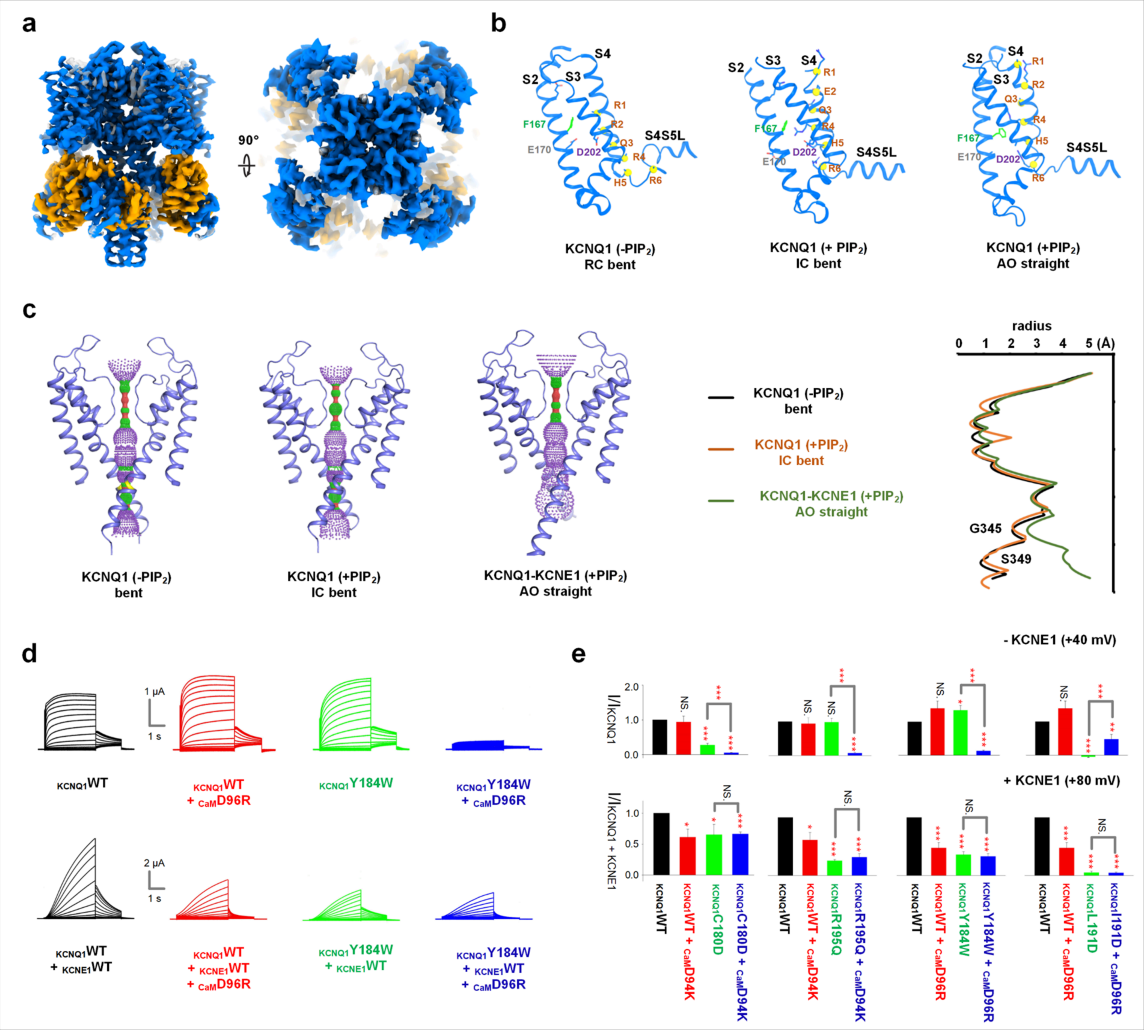
Conformations of KCNQ1 in various functional states. (**a**) Cryo-EM map and structure model of KCNQ1-E160R/R231E IC state in the presence of PIP_2_. KCNQ1, PIP_2_ and CaM are colored in blue, black and orange, respectively. (**b**) Comparison of voltage sensors between KCNQ1-RC state, KCNQ1-IC state and KCNQ1-AO state. Only the helical regions of S2-S4 and S4S5L are shown for clarity. The c-alphas pf the positive charged (or polar) resides on S4 are shown in yellow spheres with stick side chains, and the gating charge transfer center residues F167 (green), E170 and D202 are shown as sticks. (**c**) The ion conductance path and core radius for KCNQ1 bent conformation (PDB: 6uzz), KCNQ1 with PIP_2_ IC bent conformation, and KCNQ1-KCNE1 with PIP_2_ AO straight conformation, respectively. Figures are plotted using the HOLE program. (**d**) Representative current traces of one interaction pairs between KCNQ1-S2S3L and CaM: KCNQ1-Y184W and CaM-D96R, displayed without (upper) and with (lower) KCNE1 co-expression. (**e**) Current amplitude comparison (+40 mV without KCNE1, +80 mV with KCNE1) of all four identified interaction pairs, shown without (upper) and with (lower) KCNE1. The color of bars was the same as the current traces. The holding potential is −80 mV for all electrophysiology recordings. All error bars are SEM (n ≧ 10).

Voltage dependent gating of KCNQ1 involves two measurable steps of VSD activation to the I and A states, both of which trigger pore opening to the IO and AO (**Fig. S1a**). Previous structural studies indicate that KCNQ1 may adopt a bent conformation in the resting-closed (RC) state ^32^ and a straight conformation in the AO state ^11,30^. Then, what is the conformation, bent or straight, in the IO state? To address this question, we trapped the VSD of KCNQ1 in I state by introducing E160R/R231E (E1R/R2E) mutations ^24,33^ (Fig. 1a). The structure of KCNQ1-E1R/R2E in I state shares high similarity with KCNQ1-PIP_2_ structure (PDB: 9VEN), and both adopt a bent conformation: the S6 and HA helices form a helix-loop-helix structure. The R237 (R4) residue of the S4 helix points to the gate charge transfer center formed by F167, E170 and D202, while R231 (R2) and H240 (H5) of S4 helix sit within the gate charge transfer center in the resting state (PDB: 8SIN) and activated state (PDB: 9VE1), respectively. This supports that the structure is captured in the I state, with the S4 is ~5 Å lower than in the A state ^34^. The ion conductance pathway plotted using HOLE program shows that the pore radius (~1 Å at the narrowest point) is too small for hydrated potassium to pass, suggesting that the structure may represent an intermediate-closed (IC) state. In the structure, one PIP_2_ molecule is observed at the V-PIP_2_ site, which is mainly formed by the S2S3L, S3 and S4-S5 linker (S4S5L) (Figs. 1a–c).

In functional studies, while the KCNQ1-E1R/R2E channels showed constitutive macroscopic currents independent of voltage ^24,33^, single-channel recordings exhibited flickering openings with low open probability ^28^. These results are consistent with the KCNQ1-E1R/R2E structure being in the IC conformation. Then, does the IO state adopt the bent conformation? In KCNQ1 channel structures, each subunit binds a CaM molecule at the cytosolic domain^11,29,30,32,34^. In the bent conformation, CaM interacts with the S2S3L on the cytosolic side of the VSD (Fig. 1a). During VSD activation (R →I →A), if the channel remains bent, with S2S3L and CaM likely interacting differently before reaching the straight A state, these S2S3L-CaM interactions would influence activation of the IO state more than to the AO state.

To test this hypothesis, we performed mutational scanning in both CaM (D94 and D96) and S2S3L (C180, R181, S182, K183, Y184, L191 and R192) as well as in S3 (R195 and K196) to evaluate the function of individual mutations and their pairs in the activation of KCNQ1 and I_Ks_, respectively (**Figs. S2–6**). Four pairs of these mutations are noteworthy: KCNQ1-C180D in S2S3L paired with CaM-D94K and R195Q in S3 paired with CaM-D94K (Figs. 1e, **S3a, S4d**), and KCNQ1-Y184W and L191D in S2S3L, each paired with CaM-D96R (Figs. 1d, e, **S6b**). Among these four pairs of mutations, three double mutations (KCNQ1-C180D and CaM-D94K, KCNQ1-R195Q and CaM-D94K, KCNQ1-Y184W and CaM-D96R) caused near complete loss of current compared to single mutations in KCNQ1 or CaM alone, whereas the KCNQ1-L191D and CaM-D96R double mutation pair significantly recued the current of KCNQ1-L191D mutation (Figs. 1e, **S6b**). Such drastic changes in current amplitude by the double mutations suggests that these paired residues may interact during channel opening. The four mutation pairs specifically affect KCNQ1, as their double mutations in I_Ks_ produced current amplitudes similar to those of single KCNQ1 or CaM mutations (Figs. 1d, e, **S3a, S4d, S6b**). It is unlikely that these double mutations reduce surface expression, as evidenced by the robust currents exhibited by the mutant I_Ks_ complexes, although it is known that KCNQ1 and KCNE1 traffic to the plasma membrane via independent pathways in both cardiac myocytes and *Xenopus* oocytes ^35^. Since KCNQ1 opens predominantly to the IO state and I_Ks_ opens exclusively to the AO state, these results support that the channel adopts a bent conformation in the IO state and a straight conformation in the AO state. Further support for the correlation between the conformation and functional state is shown below.

## C-PIP_2_ binding is essential for channel activation

**Figure 2:**
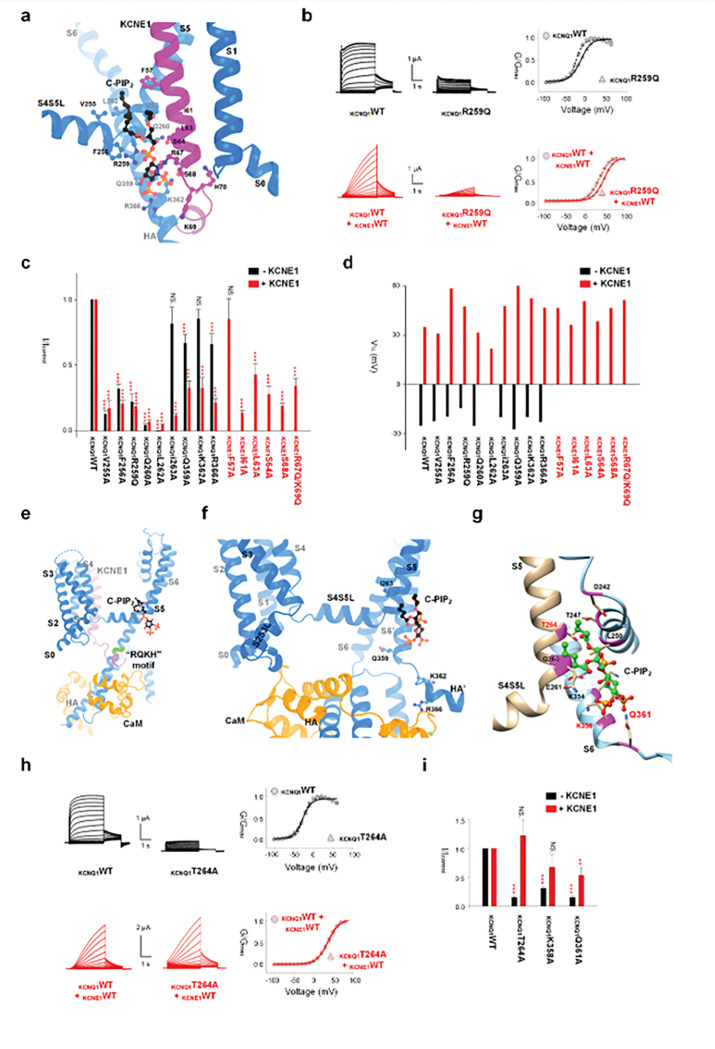
C-PIP_2_ binding is essential for channel activation. (**a**) C-PIP_2_ binding site in the KCNQ1-KCNE1-PIP_2_ complex (PDB: 9VEI). KCNQ1, KCNE1 and PIP_2_ are colored in blue, magenta and black, respectively. C-PIP_2_ is shown with ball-and-stick model. Key residues are displayed. (**b**) Representative current recordings of KCNQ1-WT (left) and mutant KCNQ1-R259Q (middle) channels, displayed without (upper) and with (lower) KCNE1 co-expression. Right: G-V relationship comparison with WT channels. (**c**) Current amplitude comparison (+40 mV without KCNE1, +80 mV with KCNE1) of mutagenesis screens of all labeled C-PIP_2_ binding sites in (**a**). The black bars show without KCNE1, and the red bars show with KCNE1. (**d**) V_1/2_ of mutagenesis screens (black for without KCNE1, red for with KCNE1) of all labeled C-PIP_2_ binding sites in (**a**). (**e**) Structure depiction of the straight conformation of one monomer of tetrameric KCNQ1-KCNE1-CaM complex (PDB: 9VEI) in the presence of C-PIP_2_. CaM and “RQKH” motif are colored in orange and green, respectively. (**f**) Molecular docking of C-PIP_2_ into the bent conformation of KCNQ1-E1R/R2E. Secondary structure of the neighboring KCNQ1 subunit are labelled with S6’ and HA’. (**g**) Structure depiction of C-PIP_2_ binding residues specific to KCNQ1 from molecular docking. (**h**) Representative current recordings of KCNQ1-WT (left) and mutant KCNQ1-T264A (middle) channels, displayed without (upper, black) and with (lower, red) KCNE1 co-expression. Right: G-V relationship comparison with WT channels. (**i**) Current amplitude comparison of C-PIP_2_ binding residue mutations identified by docking in (**g**). Black: without KCNE1, red: with KCNE1. All error bars were SEM (n ≧ 10).

In the KCNQ1-E1R/R2E structure, no obvious C-PIP_2_ was observed. Is C-PIP_2_ important for both the IO and AO states, and what role does C-PIP_2_ play? The KCNQ1-KCNE1 structure reveals that C-PIP_2_ interacts with residues in the S4S5 linker (V255 and F256), S5 (R259, Q260, L262 and L263) of one KCNQ1 subunit, and S6-HA (Q359, K362, and R366) of a neighboring KCNQ1 subunit, and the KCNE1 (F57, I61, L63, S64, R67, S68, K69, and K70) subunit (Fig. 2a). First, we mutated each C-PIP_2_-interacting residue in both KCNQ1 and KCNE1 to alanine (Ala) or neutralized charged amino acids (glutamine Q or asparagine N) and assessed the function of the mutant channels. All mutations, except KCNE1-F57A, significantly reduced I_Ks_ current amplitudes (Figs. 2b, c, **S7**). The similar effect of KCNE1-K70N was reported previously ^10^. The C-PIP_2_ site mutations also reduced KCNQ1 current amplitude in the absence of KCNE1 (Figs. 2b, c, **S7**). These results are consistent with observations made when membrane PIP_2_ levels were reduced ^10,11^, indicating that these mutations diminish PIP_2_ binding to the KCNQ1 channel and that C-PIP_2_ is critical for the conductance of KCNQ1 and I_Ks_. Importantly, all these mutations caused only minor shifts of the voltage-dependent activation, with the conductance-voltage (G-V) relationship deviating from the wild-type (WT) less than 25 mV (Figs. 2d, **S7**). This is consistent with the mechanism whereby C-PIP_2_ binding is crucial for coupling of VSD movements and pore opening but not for VSD activation ^7^.

Among the exanimated mutations, KCNQ1-I263A, Q359A, K362A and R366A produced less pronounced reductions in KCNQ1 current amplitude than the reduction observed in the I_Ks_ current (Figs. 2c, **S8a-d**). These results indicate that the C-PIP_2_ binding sites in KCNQ1 may alter in the absence of KCNE1 ^32^. Notably, residues Q359, K362 and R366 within or around the “RQKH” motif, which is the hinge for the bent and straight conformation switch (Fig. 2e), interact with C-PIP_2_ primarily in the presence of KCNE1 (Figs. 2c, **S8b-d**). Thus, the binding of C-PIP_2_ to these residues may help KCNE1 stabilize the straight conformation ^7,10^, while the disruption of the “RQKH” motif from it α-helical structure into a loop may alter C-PIP_2_ binding.

To verify this notion, we evaluated the structure of the C-PIP_2_ binding sites in the bent conformation. Since C-PIP_2_ binding was not observed in any available structures of KCNQ1 in the bent conformation, we performed molecular docking of C-PIP_2_ to the bent conformation of KCNQ1 (Fig. 2f). The results revealed that the residues I263, Q359, K362, and R366 no longer interact with C-PIP_2_ (Figs. 2f, g). These findings align with the functional data showing that mutations of these residues reduce KCNQ1 current amplitudes to a lesser extent than I_Ks_ (Figs. 2c, **S8a-d**). Molecular docking further revealed that, in the bent conformation, residues T264, K358 and Q361 interacted with the C-PIP_2_, which are not part of the C-PIP_2_ site in the I_Ks_ structure. Mutation of these residues reduced KCNQ1 current amplitudes to a greater extent than I_Ks_ (Figs. 2g–i, **S8e, f**). These results indicate that the opening of KCNQ1 channel is associated with the bent conformation, whereas the opening of I_Ks_ channel is associated with the straight conformation, and the C-PIP_2_ site alters between the two conformations. Our previous findings indicated that both KCNQ1 and I_Ks_ require PIP_2_ for channel function, but the EC_50_ of PIP_2_ dose-response for KCNQ1 was over 100-fold greater than that for I_Ks_, suggesting a higher PIP_2_ affinity for I_Ks_^10^. The differences in C-PIP_2_ binding sites within KCNQ1 across different states, combined with the role of KCNE1 in facilitating C-PIP_2_ binding, explain the >100-fold increase in PIP_2_ sensitivity observed in I_Ks_ channels compared to KCNQ1 alone. This mechanism also accounts for the observation that no PIP_2_ is resolved in the C-site in the bent conformation due to its low affinity.

## V-PIP_2_ binding is essential for channel activation to AO but not to IO

**Figure 3:**
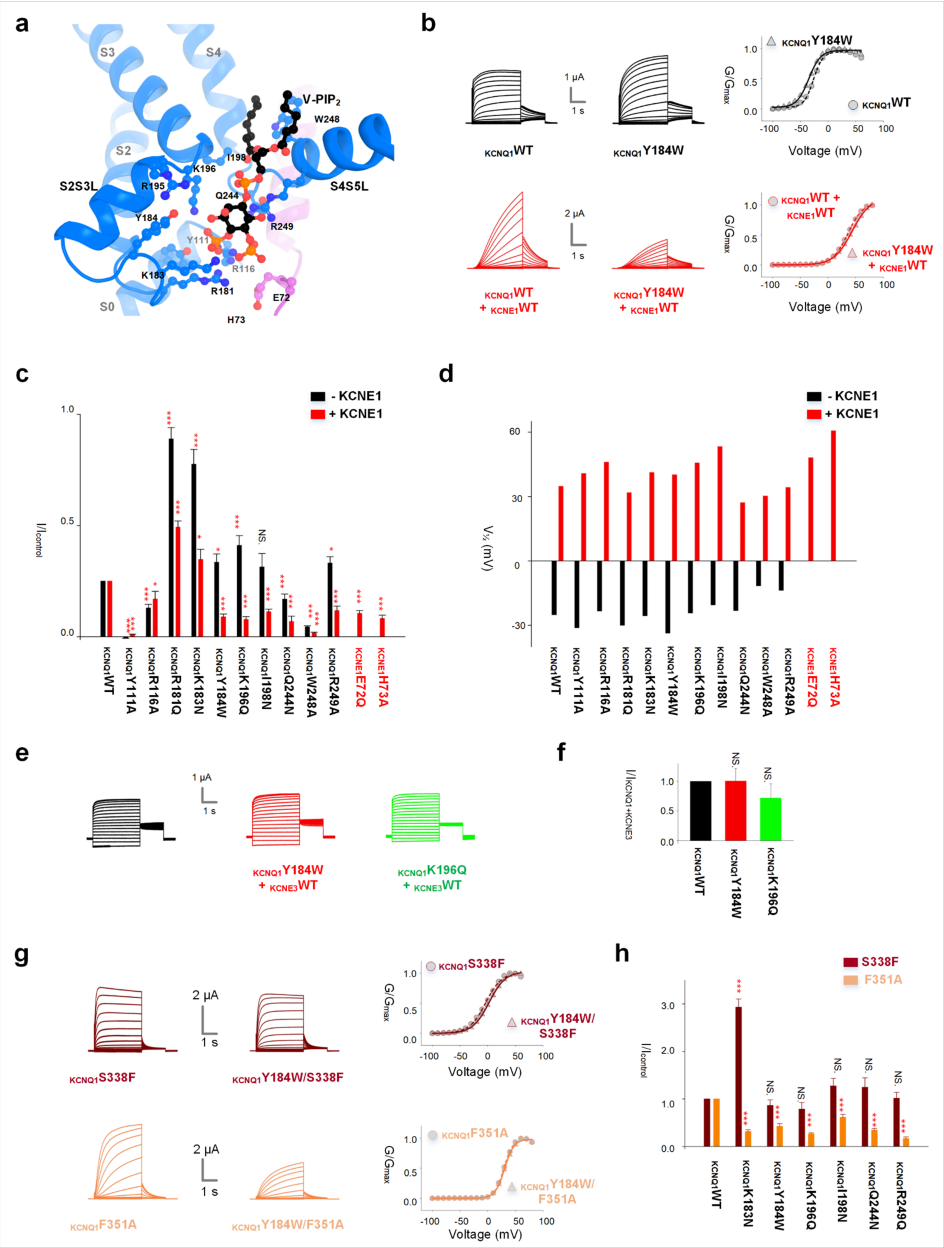
V-PIP_2_ binding is essential for channel activation to AO but not to IO. (**a**) V-PIP_2_ binding site in the KCNQ1-KCNE1-PIP_2_ complex (PDB: 9VEI). Key residues are displayed. (**b**) Representative current recordings of KCNQ1-WT (left) and mutant KCNQ1-Y184W (middle) channels, displayed without (upper, black) and with (lower, red) KCNE1 co-expression. Right: G-V relationship comparison with WT channels. (**c**) Current amplitude comparison of mutagenesis screens (+40 mV without KCNE1, +80 mV with KCNE1) of all labeled V-PIP_2_ binding sites on (**a**). The black bars show without KCNE1, and the red bars show with KCNE1. (**d**) V1/2 of mutagenesis screens (black bars: without KCNE1, red bars: with KCNE1) of all labeled V-PIP_2_ binding sites in (**a**). (**e**) Representative current recordings of KCNQ1-WT + KCNE3-WT (black), mutant KCNQ1-Y184W (red) + KCNE3-WT and KCNQ1-K196Q (green) + KCNE3-WT channels. (**f**) The current amplitudes (at +40 mV) for the respective channels. The color of bars was the same as the current traces. (**g**) Representative current recordings of KCNQ1-S338F (upper, IO only) and KCNQ1-F351A (lower, AO only), and mutant KCNQ1-Y184W on KCNQ1-S338F and KCNQ1-F351A, respectively. Right: average G-V relationship comparison for the respective channels with KCNQ1-S338F (upper) and KCNQ1-F351A (lower), respectively. (**h**) Current amplitude comparison of selected V-PIP_2_ mutants (+40 mV with KCNQ1-S338F, brown; +80 mV with KCNQ1-F351A, orange). The color of bars was the same as the current traces. All error bars are SEM (n ≧ 10).

**Figure 4:**
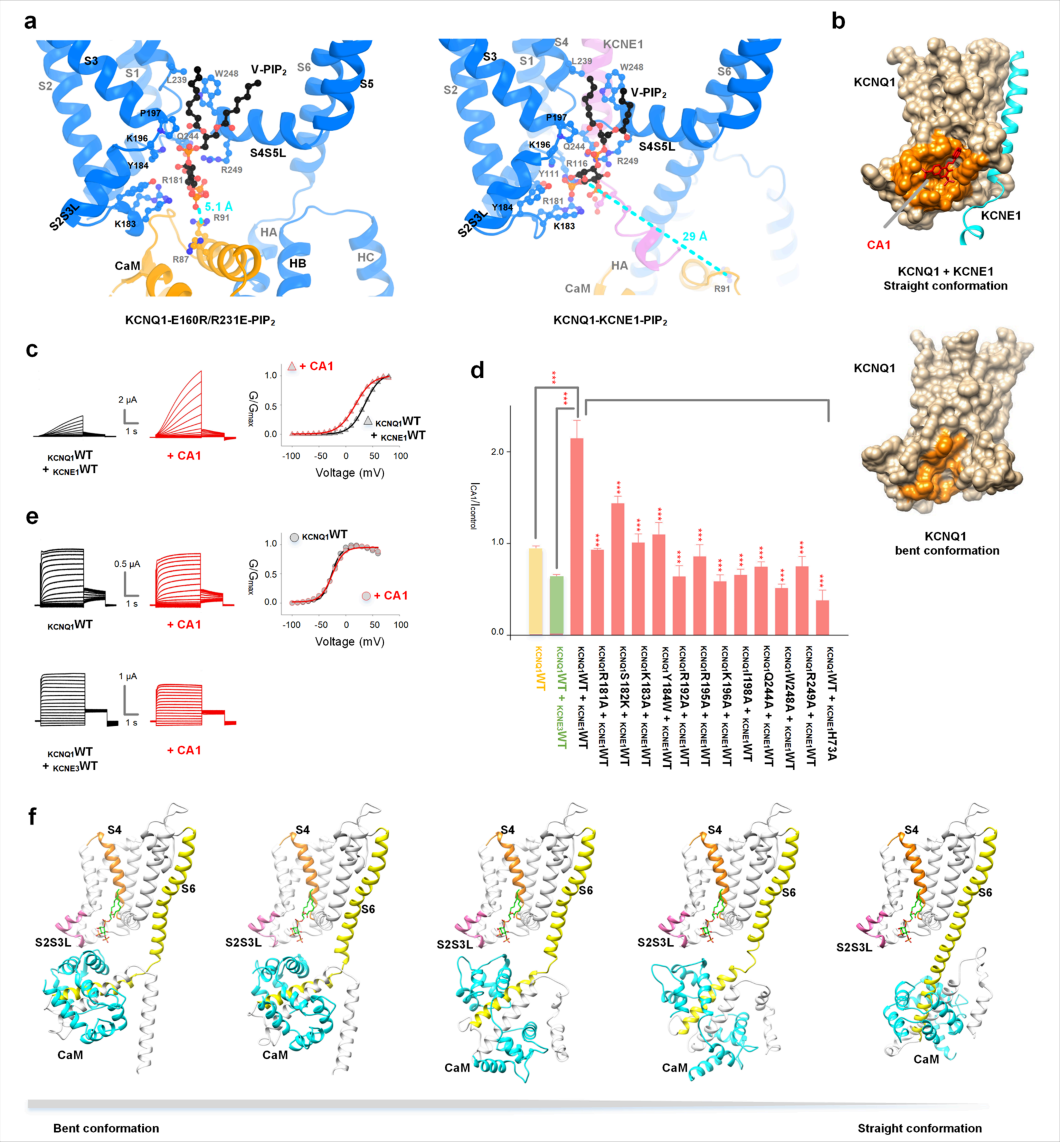
V-PIP_2_ modulates IO to AO transition. (**a**) V-PIP_2_ binding pocket with key residues displayed for KCNQ1-E1R/R2E bent conformation and KCNQ1-KCNE1 straight conformation, respectively. KCNQ1, CaM, KCNE1 and V-PIP_2_ are colored in blue, orange, magenta, and black, respectively. (**b**) *In silico* screening of CA1 to the straight conformation of KCNQ1-KCNE1 (PDB: 9VEI) and the bent conformation of KCNQ1 (PDB: 6uzz). KCNQ1, KCNE1, and CA1 are colored in tan, sky blue and red, respectively. The CA1 binding pocket is represented in orange. (**c**) CA1 effects on I_Ks_ channels. Current traces were recorded before (black) and after (red) adding 10 μM CA1. Average G-V relationship (lower) of I_Ks_ channels before (black) and after (red) adding 10 μM CA1. (**d**) Current amplitude comparative effects of CA1 on KCNQ1-WT (yellow), KCNQ1-WT + KCNE3-WT (green), I_Ks_ and putative binding residues (rose pink) from molecular docking. Changes in current amplitudes were evaluated at +40 mV without KCNE1 and at +80 mV with KCNE1. (**e**) Representative current recordings of CA1 effects on KCNQ1-WT (upper) and KCNQ1-WT + KCNE3-WT (lower). Current traces were recorded before (black) and after (red) adding 10 μM CA1. (**f**) Representative structures from the Metadynamics MD simulation illustrating the conformational transition from the bent conformation to the straight conformation (left to right). CaM is shown in cyan, V-PIP_2_ in green, S2S3L in pink, S4 in orange, and S6 in yellow.

In the structure of KCNQ1-E1R/R2E, V-PIP_2_ interacts with residues in the S2S3L (R181, K183, and Y184), the S3 (K196 and I198) and S4S5L (Q244, W248, and R249) (Figs. 1a, 4a). In the structure of KCNQ1-KCNE1 (PDB: 9VEI), V-PIP_2_ additionally interacts with the N-terminus S0 (Y111 and R116) of KCNQ1, as well as with KCNE1 (E72 and H73) (Fig. 3a). We mutated these V-PIP_2_ interacting residues to A or N/Q using a mutation scanning. Most mutations in KCNQ1 (R181, K183, Y184, K196, and R249) did not reduce but rather increased KCNQ1 current amplitudes (Figs. 3c, **S9c-f, i**), with the exceptions of Q244N and W248A (Figs. 3c, **S9g, h**), which are critical for VSD-pore coupling ^25^. Y111A and R116A reduced KCNQ1 current amplitudes (Figs. 3c, **S9a, b**); however, these residues did not interact with V-PIP_2_ in the bent conformation and the IO state, indicating that the effects of Y111A and R116A on KCNQ1 current amplitudes may not directly disrupt V-PIP_2_ binding. On the other hand, most of the mutations significantly reduced the current amplitudes of I_Ks_ except for the KCNQ1-R181Q + KCNE1 and KCNQ1-K183N + KCNE1 (Figs. 3c, **S9c, d**). Although these two mutations enhanced I_Ks_ current amplitudes, the enhancement was significantly smaller compared to the enhanced KCNQ1 current amplitudes of the same mutant. All mutations induce small GV shifts in both KCNQ1 (less than 15 mV) and I_Ks_ (less than 25 mV) (Figs. 3d, **S9**), indicating that the mutations did not modify current amplitudes by altering voltage dependence. Additionally, we observed that mutations at the V-PIP_2_ sites did not significantly affect the current amplitudes of KCNQ1 + KCNE3 (Figs. 3e, f), whereas mutations at the C-PIP_2_ sites drastically reduced the current amplitudes of KCNQ1 + KCNE3 (**Fig. S10**). Thus, these results suggest that V-PIP_2_ is essential for the AO state but not the IO state, as both KCNQ1 and KCNQ1 + KCNE3 primarily open to the IO state, whereas I_Ks_ exclusively opens to the AO state ^24,25,28,34^.

To validate this mechanism, we introduced some of the V-PIP_2_ site mutations into the mutant KCNQ1-S338F and KCNQ1-F351A, which were previously established as opening only in the IO and AO state, respectively ^24,28^. Notably, these mutations either had no impact (Y184, K196, I198, Q244, and R249) or enhanced KCNQ1-S338F current amplitudes (K183), while significantly reducing KCNQ1-F351A current amplitudes (Figs. 3g, h, **S11a-d**), supporting the notion that V-PIP_2_ is specifically required for the AO state, but not the IO state. By contrast, KCNQ1-R259Q, which affects C-PIP_2_ binding, reduced the current amplitudes of both KCNQ1-S338F and KCNQ1-F351A (**Fig. S11e**).

## V-PIP_2_ modulates IO to AO transition

Comparing V-PIP_2_ and its binding site in the structures of KCNQ1-E1R/R2E and KCNQ1-KCNE1 (Fig. 4a), notable differences were observed in the interaction network of PIP_2_. In the I state, the head group of PIP_2_ contacts S4, S4S5L, S2S3L and CaM. Upon further activation of the VSD, S4 moves upward, followed by S4S5L and S2S3L, a motion that appears to elevate the center of the PIP_2_-binding pocket. This motion weakens interactions between CaM and the rest of the V-PIP_2_ site, facilitating the alteration of the V-PIP_2_ site and repositioning of V-PIP_2_ to form closer interactions with S2S3L (Figs. 4a, **S12**). The upward movement of the PIP_2_-interaction network, the detachment of CaM from the V-PIP_2_ site, and the alteration of V-PIP_2_ binding are associated with the “bent-to-straight” conformational transition of the KCNQ1 channel.

To validate the observed structural difference in V-PIP_2_ binding between the I and A states, we employed small-molecule screening approaches ^36^. We reasoned that a compound selectively binding to the V-PIP_2_ site in the straight conformation would modulate the I_Ks_ channel without affecting KCNQ1 alone in the I state. We screened the Available Chemical Database (ACD, Molecular Design Ltd.) by docking its compound to the V-PIP_2_ binding sites of KCNQ1-KCNE1in the straight conformation. Docking results revealed that the compound CA1 (**Fig. S13a**), selectively binds to the straight conformation of the channel, with the binding site nearly overlapping with the V-PIP_2_ site. In contrast, the binding pocket undergoes structural rearrangement in the bent conformation, preventing CA1 from binding (Fig. 4b).

Our functional data showed that CA1 significantly enhanced the current amplitudes of I_Ks_ and shifted its GV to more negative voltages (Fig. 4c). Mutations in the putative CA1-binding residues (**Fig. S13b**) attenuated the effects of CA1 (Figs. 4d, **S13d-o**), supporting our docking results for the CA1 binding site in the straight conformation of the I_Ks_ channel, which opens exclusively to the AO state. Conversely, CA1 exhibited no enhancing effect on channels predominantly open to the IO state in the bent conformation, including KCNQ1 alone and the KCNQ1-KCNE3 complex (Figs. 4d, e). Notably, KCNQ1-KCNE3 could adopt a straight conformation and open in the AO state, albeit with a low probability ^30^. Docking of CA1 onto the straight conformation of KCNQ1-KCNE3 showed that CA1 could bind to the same pocket (**Fig. S13c**). Thus, the absence of CA1-induced current augmentation in KCNQ1 + KCNE3 channels is consistent with the complex primarily conducting in the IO state via the bent conformation, which precludes CA1 binding.

To support the above mechanism that the VSD movement to the A state and the change in V-PIP_2_ trigger the bent to straight conformational switch, we performed molecular dynamics (MD) simulations by first constructing a hybrid starting model. In this model, the VSD and pore domains (residues 1–236) preceding the bending point (RQKH, residues 251–254), including the bound PIP_2_, were taken from the straight conformation, whereas the remaining regions were retained from the bent conformation. We then performed metadynamics simulations to accelerate sampling along the “bent-to-straight” transition. Representative snapshots are shown in Fig. 4f. Along the biased trajectory, CaM progressively dissociated from the VSD and rotated while KCNQ1 spontaneously adopted a straight configuration. These conformational changes align with our structures of the I and A states and support the mechanism in which upward S4 movement facilitates VSD activation and conformational changes in the V-PIP_2_ binding site, displacing CaM and stabilizing a straight cytosolic conformation, thus promoting the AO state.

To visualize the conformational transition from the bent conformation to the straight conformation, we also performed targeted MD simulations. Snapshots from the simulation are presented in **Fig. S14 and Movie. S1**. During the transition, the S4 helix exhibits an upward movement compared to its position in the bent conformation (**Fig. S14a**). The phosphatidylinositol head group of V-PIP_2_ undergoes a reorientation toward S2S3L, establishing contacts with this linker (**Fig S14b**). Concurrently, CaM gradually dissociates from the VSD.

## A model of the activation of KCNQ1 and I_Ks_ by VSD, PIP_2_ and CaM

**Figure 5:**
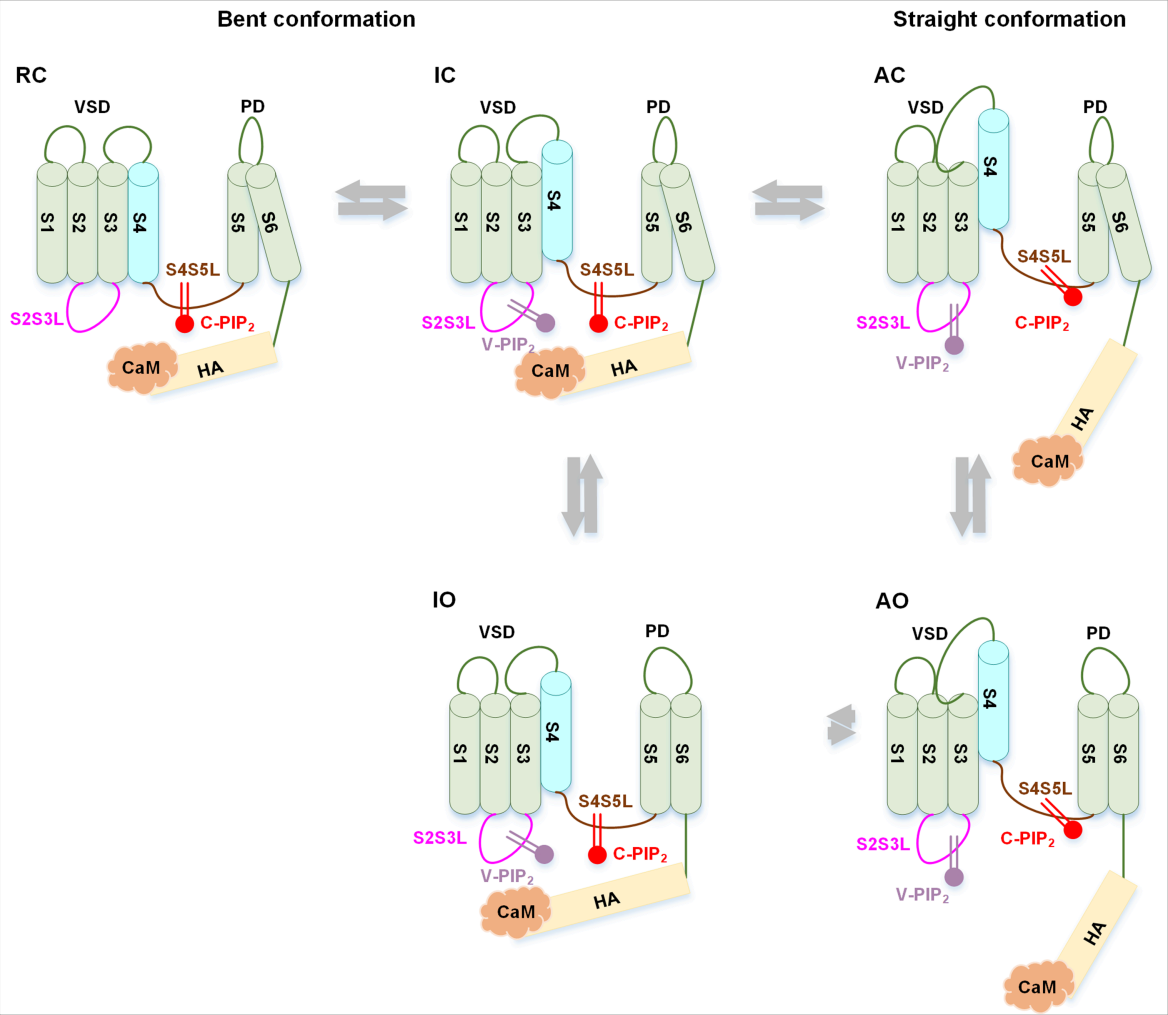
A model depicting the activation of KCNQ1 by VSD, C-PIP_2_, V-PIP_2_ and CaM. In this model, KCNQ1-RC, IC and IO states adopt a bent conformation, while the AC and AO states adopt a straight conformation. V-PIP_2_ associates with the channel during activation of the VSD to the I state, including IC and IO, through a voltage-dependent mechanism that triggers the IO-AO transition. CaM and V-PIP_2_ compete for binding to the S2S3L; when CaM binds, the channel adopts a bent conformation, whereas binding of V-PIP_2_ results in a straight conformation. C-PIP_2_ interacts with the channel in both the I and A states, facilitating the coupling of VSD and pore during the IC-IO and AC-AO transitions.

Based on our findings and prior studies, we propose a model for KCNQ1 and I_Ks_ activation (Fig. 5). In this model, V-PIP_2_ interacts with the channel via a voltage-dependent mechanism to trigger the IO to AO transition, while C-PIP_2_ is associated with the channel to mediate the VSD-pore coupling and stabilize the straight conformation. The recently published structure of KCNQ1 with the VSD in the RC state indicated that the channel adopts a bent conformation, which obstructs the V-PIP_2_ site and prevents its binding ^32^. Therefore, V-PIP_2_ binds to the channel upon VSD activation to the I state, in both IC and IO, which adopt the bent conformation (Fig. 5). V-PIP_2_ is subsequently reoriented upon VSD further activation to the A state, triggering the switch from bent to straight, which culminates in the activating-closed (AC) and AO states (Figs. 3, 4f). C-PIP_2_ binds to the channel in both the I and A states to facilitate the VSD-pore coupling during the IC-IO and AC-AO transitions, but whether C-PIP_2_ binds to the RC state is not clear. In the I state, C-PIP_2_ binds to the channel with a lower affinity than in the A state owing to the alteration of the C-PIP_2_ binding sites during the “bent-to-straight” transition (Fig. 2). The KCNE1 association inhibits the IO state and enhances the AO state (**Fig. S1a**) ^24,25,28^.

We propose that, even in association with KCNE1, the KCNQ1 subunit undergoes the same bent and straight transition as the VSD moves from the I state to the A state, based on the following observations. First, V-PIP_2_ is essential for the activation of the KCNQ1 + KCNE1 channel (Fig. 3), potentially by stabilizing the straight conformation. Second, C-PIP_2_ in I_Ks_ exhibits a state-dependent change in binding affinity; in the open state (AO, straight), the affinity is enhanced, and C-PIP_2_ is resistant to digestion by the lipid phosphatase CiVSP. By contrast, the C-PIP_2_ affinity decreases following channel deactivation at hyperpolarized voltages (IC and RC, bent), allowing for digestion by CiVSP ^37,38^.

The correspondence between RC, IC, and IO (all adopting bent conformation) and AO (adopting the straight conformation) is supported by structural and functional evidence from previous studies ^24,25,28–31,34^ and from this study. However, whether the AC state corresponds to the straight conformation is not supported by available data and remains an assumption in the model. This model accounts for many aspects of KCNQ1/ I_Ks_ channel function and underscores the pivotal roles of PIP_2_ in their gating mechanism.

Compounds, such as CA1, which target the V-PIP_2_ binding site modulate the activity of I_Ks_ channel (Fig. 4b–e) and may specifically modify cardiac physiology without affecting other tissues, such as epithelium, due to the tissue-specific distribution of KCNE1. This feature potentially aids in the development of safe and effective antiarrhythmic therapies.

## Methods

### Constructs and mutagenesis

Point mutations were introduced in KCNQ1, KCNE1, and CaM via overlap extension and high-fidelity polymerase chain reaction. DNA sequencing verified the existence of all introduced mutations. Mutant complementary RNA (cRNA) was synthesized using the mMessage T7 polymerase kit (Applied Biosystems-Thermo Fisher Scientific). cRNA stocks were stored at −80 °C.

### Cryo-EM sample preparation and data collection

Construct design, protein expression and purification, nanodisc reconstitution for KCNQ1-KCNE1 followed the same protocol as the recent study ^11^. Briefly, N- and C-terminus loop of KCNQ1 are truncated for stability, resulting in a construct including residues 76–620. I145C and K41C mutations were introduced to KCNQ1 and KCNE1, respectively, for KCNQ1-KCNE1 stabilization and purification. To trap KCNQ1-VSD into I state, E160R (E1R) and R231E (R2E) were introduced to KCNQ1. Viruses of KCNQ1-I145C-E1R-R2E and KCNE1-K41C were added with volume ratio 1:1. Resolved structure lacks KCNE1, but CaM binds to KCNQ1 at a 1:1 stoichiometry, as an essential structural component.

Quantifoil R1.2/1.3 (400 mesh) holey carbon gold grids were glow-discharged for 30-s. The concentrated protein sample was mixed with 120 mM Fos-Choline-8 at a volume ratio of 1:50 immediately before applying to the grid. 3.5 μL of ~2.5 mg/mL protein sample was applied to each grid, after 20-s waiting, which were double-blotted for 5-s under blot force −3 at 100% humidity and 16 °C, then vitrified by plunging into liquid ethane cooled by liquid nitrogen using Vitrobot Mark IV (FEI).

Dataset was acquired on 300 keV Titan Krios microscope (FEI) equipped with a K3 direct electron detector (Gatan) using EPU software with magnification of 130,000. Data collection was conducted in super-resolution mode with pixel size of 0.6485 Å. Images were recorded with a defocus range of −1.0 to −2.0 μm at a dose rate of 14.7 e/frame/s with images captured over 50 frames.

### Cryo-EM image processing and 3D reconstruction

Image stacks were gain-normalized and corrected for beam-induced motion using MotionCor2 ^39^. The contrast transfer function parameters were estimated from motion-corrected summed images without dose-weighting using CTFFIND4 ^40^. All subsequent processing steps were performed on motion-corrected, dose-weighted summed images. Data processing was performed in CryoSPARC ^41^. 2D classification was conducted to remove junk particles. Good particles were subjected to Ab-Initio Reconstruction and Heterogeneous Refinement with C1 symmetry. Correct conformational particles were sorted out and input to Non-Uniform Refinement to generate a final reconstruction map with C4 symmetry.

### Cryo-EM structural refinement and model building

Initially, KCNQ1 bent (PDB 6UZZ) was docked into the cryo-EM map KCNQ1-I145C-E160R-R231E. Model was manually built in Coot ^42^. PIP_2_ molecule was generated as CIF file by the phenix.eLBOW ^43^ and imported as PT5. The structural model was iteratively refined using phenix.real_space_refine ^44^ with secondary structure restraints and checked in Coot. The quality of the structures was assessed using the MolProbity server ^45^.

The pore radii were calculated using HOLE ^46^. Figures were created using PyMOL (The PyMOL Molecular Graphics System, Version 2.6.0 and 3.1.1, Schrödinger, LLC) and UCSF Chimera ^47^.

### Molecular docking

The interaction between C-PIP_2_ and KCNQ1 in the bent conformation (Figs. 2f, g) was modeled using AutoDock Vina ^48^. The docking box was set to include residues surrounding the “RQKH” motif, and the side chains of residues Q360, K354, K358, and Q361 at the C-PIP_2_ binding site were treated as flexible during docking. It is noteworthy that PIP_2_ has two long and highly flexible fatty acid chains, which pose challenges for molecular docking. To address this, we used a PIP_2_ structure with shortened tails (each containing four carbon atoms) for docking. The PIP_2_ structure was treated as flexible. The exhaustiveness parameter was increased from the default value of 8 to 64. A predicted model of the C-PIP_2_-KCNQ1 complex in the bent conformation is consistent with mutagenesis results (Figs. 2g–i, **S8e, f**).

### *In silico* compound screening

The *in silico* screening strategy employed our recently developed template-guided docking method ^36^ as the search engine to screen a subset of the Available Chemical Database (ACD, Molecular Design Ltd.), consisting of approximately 10,000 compounds. Each compound in this subset carries two formal charges and was screened against the V-PIP_2_ binding site in the KCNQ1-KCNE1 complex structure.

For each compound in the chemical library, OMEGA2 (Version 3.0.1.2, OpenEye, Cadence Molecular Sciences, Santa Fe, NM, USA, http://www.eyesopen.com/) ^49,50^ was used to generate up to 200 conformers. These 3D conformers were then superimposed onto the co-bound V-PIP_2_ (focused on the head group) using the 3D similarity calculation program SHAFTs ^51^. Subsequently, the molecular docking program AutoDock Vina ^48^ was employed to refine the complex structures generated during the superposition step. Compounds were ranked using a hybrid scoring function implemented in our method. Briefly, the ranking score combines the 3D similarity between each compound and the template PIP_2_ with the binding score of the compound at the V-PIP_2_ site. Additional details of our template-guided docking method are provided in Ref. ^36^. The predicted binding modes of the top-ranked compounds were further evaluated by visual inspection. Given that the V-PIP_2_ binding site contains several positively charged residues (e.g., R181, K183, and K196), we prioritized compounds with negatively charged groups capable of forming salt bridges with these residues.

As a result, compound CA1 was identified as an active binder of KCNQ1 + KCNE1 at the V-PIP_2_ site in experimental assays (Fig. 4c).

### Metadynamics simulations

We examined the conformational transition of the KCNQ1 channel from the bent to straight conformation without applying external steering forces, as is done in targeted MD (see the next subsection). Well-tempered metadynamics simulations were performed to accelerate this transition using Amber22 ^52^ patched with PLUMED version 2.9.3 ^53^. Simulations were carried out using a single subunit of KCNQ1 in complex with CaM. The initial hybrid structure was constructed by combining the VSD and pore domains (residues 1–236) preceding the bending point (“RQKH”, residues 251–254), including the bound PIP₂, from the straight conformation, with the remaining regions taken from the bent conformation. The system was embedded in a POPC bilayer and solvated with TIP3P water and 0.15 M NaCl using the CHARMM-GUI server ^54^. PIP_2_ structures were optimized at the HF/6–31G** level using the Gaussian 16 package ^55^, followed by single-point energy calculations at the B3LYP/cc-pVTZ level to obtain the electrostatic potential (ESP). Restrained ESP (RESP) charges were derived from these calculations for use in force field parameterization. Protein parameters were assigned using the AMBER ff14SB force field ^56^, and ligand parameters were generated using the general AMBER force field 2 (GAFF2) ^57^. The collective variable (CV) was defined as the RMSD of KCNQ1 relative to the straight conformation, and well-tempered metadynamics biasing was applied along this CV to enhance sampling of the “bent-to-straight” transition.

### Targeted molecular dynamics (TMD) simulations

TMD simulations were performed to visualize the conformational transition of the KCNQ1 channel from the IO state (initial structure) to the AO state (target structure). TMD applies a biasing potential that drives the system from an initial to a target conformation by minimizing the root-mean-square deviation (RMSD) between selected atoms of the current and target structures over the course of the simulation. To reduce system complexity and computational cost, we performed the simulations using a single subunit of KCNQ1 in complex with CaM in an implicit solvent system, rather than the full tetrameric complex embedded in a lipid bilayer. This simplification is justified because both the IO and AO conformations have been experimentally determined, and the major structural differences occur at the single-chain level. Our TMD simulation goal was to visualize the conformational changes within one KCNQ1-CaM unit. In our setup, the Cα atoms of the AO state conformation were defined as the target reference. A force constant of 100 kJ·mol^−1^·nm^−2^ was applied to drive the transition. The initial and target structures were pre-aligned prior to simulation to remove overall translational and rotational differences. Artificial rotational motion was further removed every 10 simulation steps to ensure smooth convergence toward the target. TMD simulations were carried out using the Amber22 software suite patched with PLUMED version 2.9.3.

### Ion channel expression in *Xenopus* oocytes

Stage *V* or *VI* oocytes were procured from *Xenopus* laevis via laparotomy, in accordance with the protocol approved by the Washington University Animal Studies Committee (protocol #24–0405). Oocytes were subjected to digestion with collagenase (0.5 mg/ml, sigma-Aldrich, St. Louis) and subsequently injected with channel cRNAs using a Nanoject (Drummond, Broomall). Each oocyte was injected an identical amount of cRNAs corresponding to either WT or mutant KCNQ1. In tests using KCNE1 or CaM co-expression, KCNE1 and CaM cRNAs were co-injected at mass ratios of 4:1 (KCNQ1: KCNE1) and 1:1 (KCNQ1: CaM), respectively. Injected oocytes were incubated in ND96 solution [96 mM NaCl, 2 mM KCl, 1.8 mM CaCl_2_, 1 mM MgCl_2_, 5 mM Hepes, 2.5 mM CH_3_COCO_2_Na, and 1: 100 penicilin-streptomycin (Ph 7.6)] at 18 °C for 3–5 days before recording.

### Two-electrode voltage clamp (TEVC)

Microelectrodes were fabricated using thin wall borosilicate glass (B150–117-10) and a micropipette puller (P-1000, Sutter Instrument, Novato, CA). The pipette resistance ranged from 0.5 to 3 MΩ when filled with a 3 M KCl solution and immerged in ND96 solution, which comprises 96 mM NaCl, 2 mM KCl, 1.8 mM CaCl_2_, 1 mM MgCl_2_, 5 mM Hepes, and 2.5 mM CH_3_COCO_2_Na at pH 7.6. The experiments were recorded in ND96 solutions at room temperature. Whole-cell currents were recorded using a GeneClamp 500B amplifier (Axon Instruments, CA) driven by Patchmaster (HEKA, Holliston, MA). To prevent aliasing, the device applied a low-pass filter to the measured currents at 2 kHz.

The testing voltages were from −100 mV to +60 mV (without KCNE1) or +80 mV (with KCNE1 or KCNQ1-F351A) with 10 mV increments and then returned to −40 mV for measuring the tail currents. The holding potential is −80 mV for all electrophysiology recordings.

In compound CA1 application experiments, CA1 was initially dissolved in Dimethyl Sulfoxide (DMSO) to make a 10 mM stock solution, and 1 μL of this stock was added to 1000 μL bath solution (ND96) to achieve a final concentration of 10 μM using a manual pipette. The recording chamber was thoroughly rinsed with 70% ethanol and the deionized water following each experiment involving CA1 administrations.

All the electrophysiological recordings were repeated with at least two different batches of oocyte.

### Electrophysiological data analysis

Data were analyzed using MATLAB (MathWorks, MA) and Sigmaplot (SPSS) software. The G-V relationship calculation involved normalizing the instantaneous tail currents subsequent to test pulses to the maximum current. The G-V relationship was fitted using a single Boltzmann equation in the form of

$$G(V)=(1+exp{\left(-V_{s}\left(V-V_{1/2}\right)\right)}^{-1}$$

where V represents the test pulse voltage, $V_{1/2}$ represents the half-activation voltage, and $V_{s}$ regulates the steepness of the Boltzmann equation. $V_{s}$ is related to RT/zF, where R denotes the gas constant, T represents the temperature, z signfies the equivalent valence, and F indicates the Faraday constant. The current amplitude comparison was determined using the steady-state current amplitude at the end of the four-seconds test pulse.

### Statistical analysis

Data was presented as mean ± standard error of mean (SEM), with n specifying the number of independent experiments. Statistical analysis, including t-test and one-way ANOVA, were performed using SPSS. Statistical significance was designated as “*” for p ≦ 0.05, “**” for p ≦ 0.005, and “***” for p ≦0.0005; “NS.” represents no statistical difference.

## Supplementary Material

Supplementary Files

This is a list of supplementary fi les associated with this preprint. Click to download.

• FigureS13.tif

• FigureS8.tif

• FigureS10.tif

• FigureS5.tif

• MovieS1.mp4

• FigureS14.tif

• FigureS11.tif

• FigureS1.tif

• FigureS4.tif

• FigureS6.tif

• FigureS3.tif

• FigureS9.tif

• FigureS2.tif

• FigureS7.tif

• FigureS12.tif

• Supplementaryinformation.docx

Supplementary Information is available for this paper.
